# Annotations

**Published:** 1887-05-21

**Authors:** 


### ANNOTATIONS.

**The Vienna Hospital Scandal.** THE Allgemeines Krankenhaus, the great Vienna hospital, has just attracted public attention, followed by an outburst of public indignation which seems likely to compel the retirement of the whole of the present administration. It is declared that the patients have been badly nursed, underfed, and cruelly treated. Indeed, no patient who was poor ever received adequate care or attention, or even food, because, forsooth, his poverty forbade his bribing the nurses and other officials. We are not surprised at these revelations, because we have known for some years that it was impossible for the Government, or indeed for anyone, to procure precise, or indeed any reliable information of the working of this institution. Wherever this is the case—and we regret to say it is the case in respect to three general hospitals in London dependent on voluntary contributions—the public will do well to withhold their support, on the ground that where officials and committees decline or withhold information, there it is reasonable to believe, and usually found to be true, that bad management, extravagance, or worse evils exist to a relatively large extent. These three general hospitals, like the Vienna Allgemeines Krankenhaus, would be better for a thorough investigation and inquiry. Two, at least, published figures which are open to question, and all require in the public interest a radical change in the existing methods of administration. In Vienna, where the Government control the public institutions to an extent unknown in this country, a decree was published on Friday ordering a most circumstantial inquiry, the results of which must prove beneficial to the patients at any rate. In London, in the three general hospitals referred to, the abuses are no doubt less apparent than in Vienna, and we have no redress beyond that which the quickening of public opinion, followed by pressure upon the committees of management, may ultimately produce.

**A Book of Grievances.** One result of the discovery of the ill-treatment and neglect of patients in the Vienna Hospital has been to induce the Statthalter of Lower Austria to enact that in all public hospitals a Book of Grievances shall be kept, and periodically laid before him, in which patients may write their complaints before leaving the hospital. We think there is much to be said in favour of the universal adoption of such a system. It would place it beyond the power of a grumbler to leave the hospital with thanks on his lips and discontent at his heart, as in the case we recently referred to. It would give to the well-managed hospitals a lever of convincing force with which to raise funds from those whose sympathies are not aroused by general statements. It would put the officials on their mettle, and make the patients careful in their statements, and so produce results of great practical importance. The action of the Austrian Statthalter has been anticipated in this country by the Committee of the Home Hospitals Association for Paying Patients. Fitzroy House, Fitzroy Square, the first English pay hospital, has treated many hundreds of the middle and upper classes in this country, and, as a result of their experiences, the committee, on the motion of Lord Aberdeen, determined a few years ago to prepare a form which every patient should sign on leaving the institution, and in which an expression of their views of the management and the treatment they received was to be entered. The result has been in every way satisfactory, and the general adoption of the system by hospitals all the world over could not fail to prove a useful and helpful innovation.

**The Registration of Trained Nurses.** Nursing, as a profession, has become an important fact, not only to thousands of women, but to the public at large. Many complaints are made that so-called nurses still find their way into private houses and families, and there disport themselves after the manner of Sairey Gamp and her sisters. The continuance of such a possibility is as harmful to the trained nurse as it is dangerous to the public health. In these circumstances the action of the Council of The Hospitals Association in deciding to establish a Register of Trained Nurses, and to issue a badge, is a matter of no little importance. Any nurse will be placed on the register who can furnish satisfactory proofs that she has worked for at least one year on the staff of one hospital or infirmary, and that she has been trained in the duties of a nurse. A certificate or testimonial of good moral or general conduct from the matron or lady superintendent of the hospital in which she has been trained, and the payment of an entrance-fee of half-a-crown, to include the cost of the Association's badge, and an annual fee of one shilling afterwards, are the conditions of registration, which have been carefully prepared by the Nursing and Domestic Management Committee of The Hospitals Association. Each nurse will receive a card, to be produced whenever requested, containing the address of The Hospitals Association, to whom each registered nurse can refer for testimonials to character; whilst the public will be asked to communicate their experiences of her conduct to the same authority. The heads of households and others requiring the services of a trained nurse will be able to secure a guarantee of efficiency and good conduct, by engaging one who wears the Association's badge, and such nurses will no doubt find their services at a premium. Any nurse proved to be guilty of intemperance, other serious misconduct, or neglect of professional duties, will be struck off the register after due inquiry. So much good is likely to result from this scheme, that we are surprised that no steps have been taken previously to establish a register for trained nurses. All communications should be addressed to the Secretary, The Hospitals Association, Norfolk House, Norfolk Street, London, W.C

